# Supplementary material for: Evidence of human infection by a new mammarenavirus endemic to Southeastern Asia
Source: eLife. 2016 Jun 9;5:e13135. doi: 10.7554/eLife.13135 (PMC4900801; doi:10.7554/eLife.13135)

**Table 2-source data 1: PASC analysis**

C0617: L segment


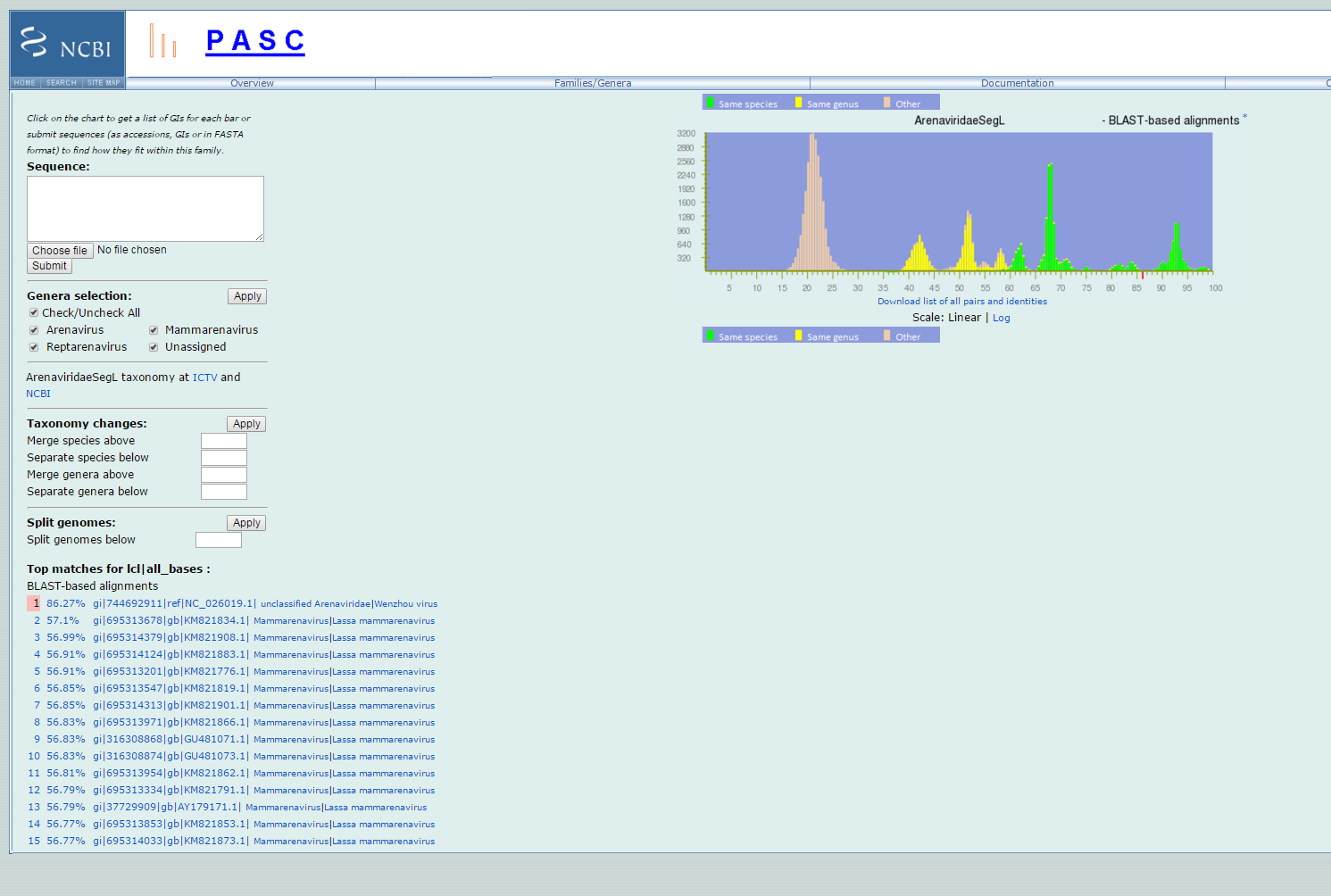


C0617: S segment


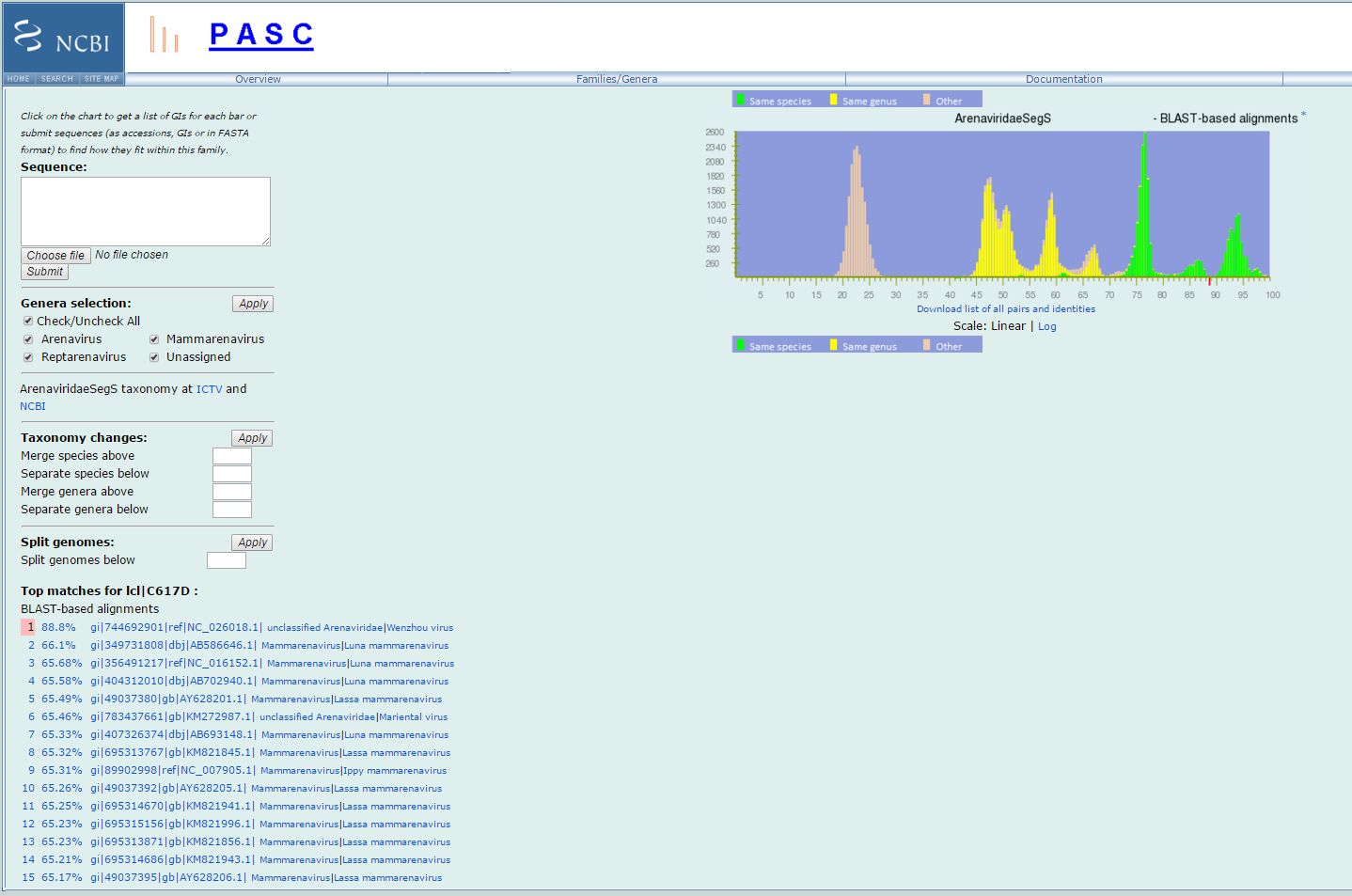


C0649: L segment


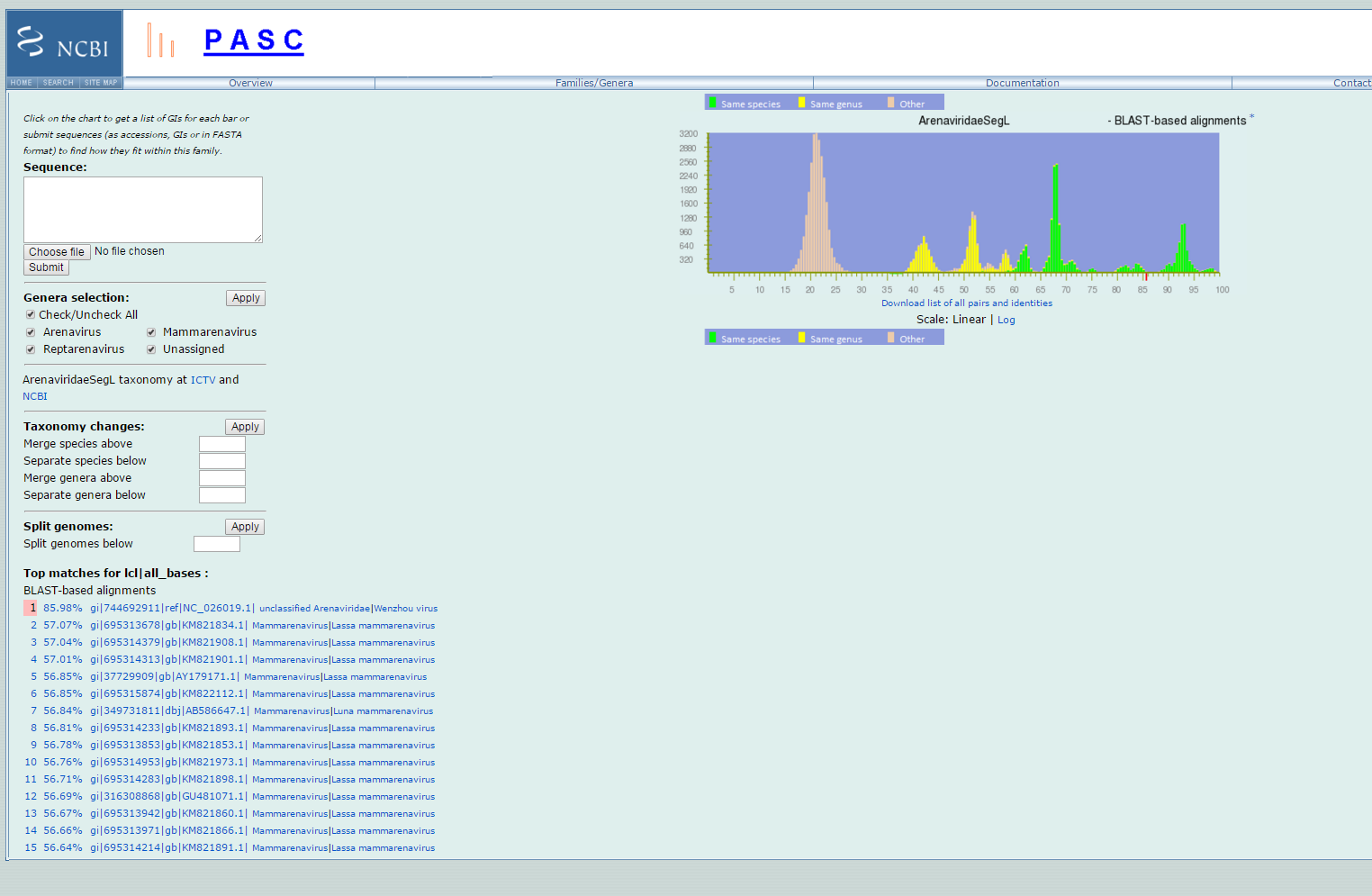


C0649: S segment


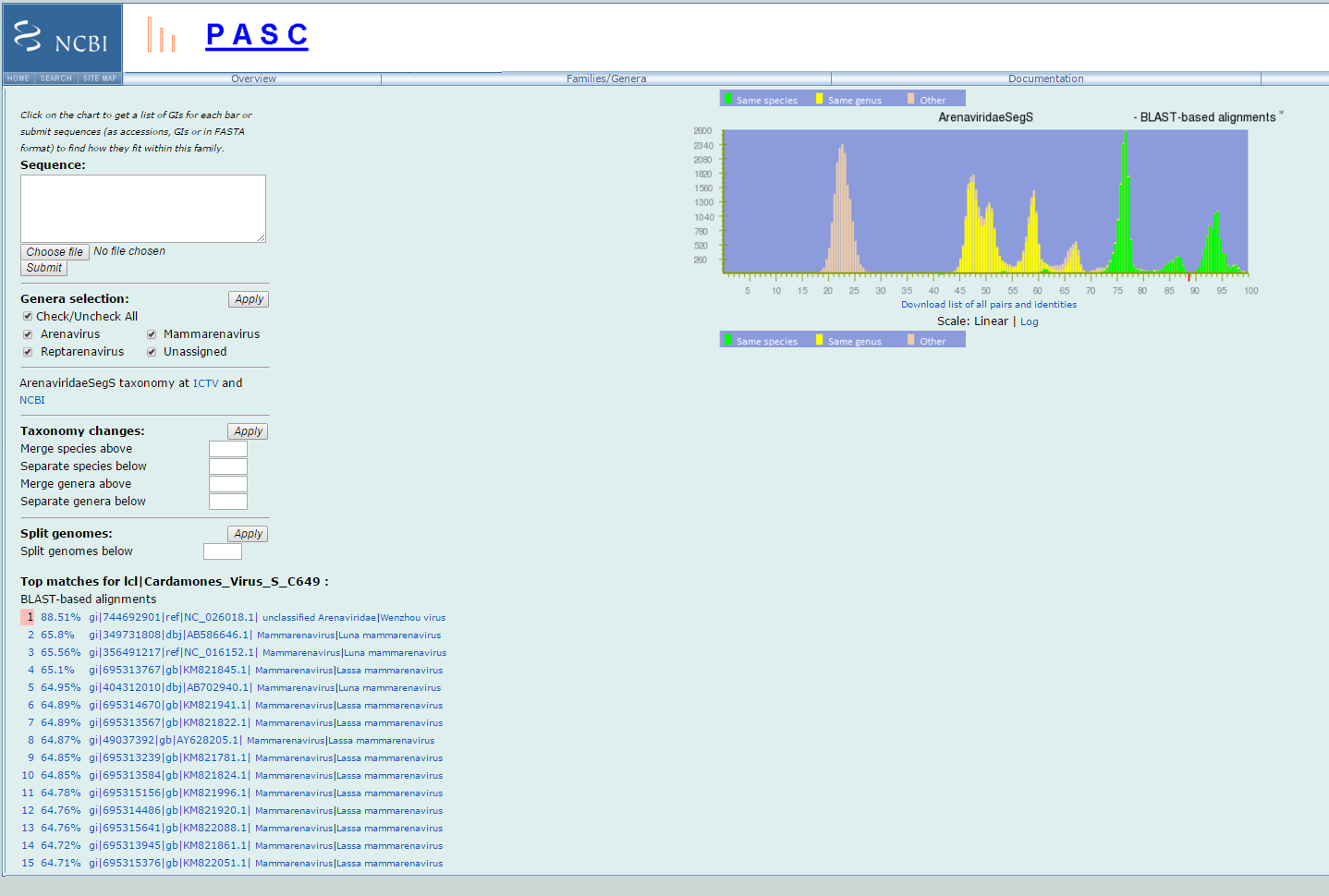


R4937: L segment


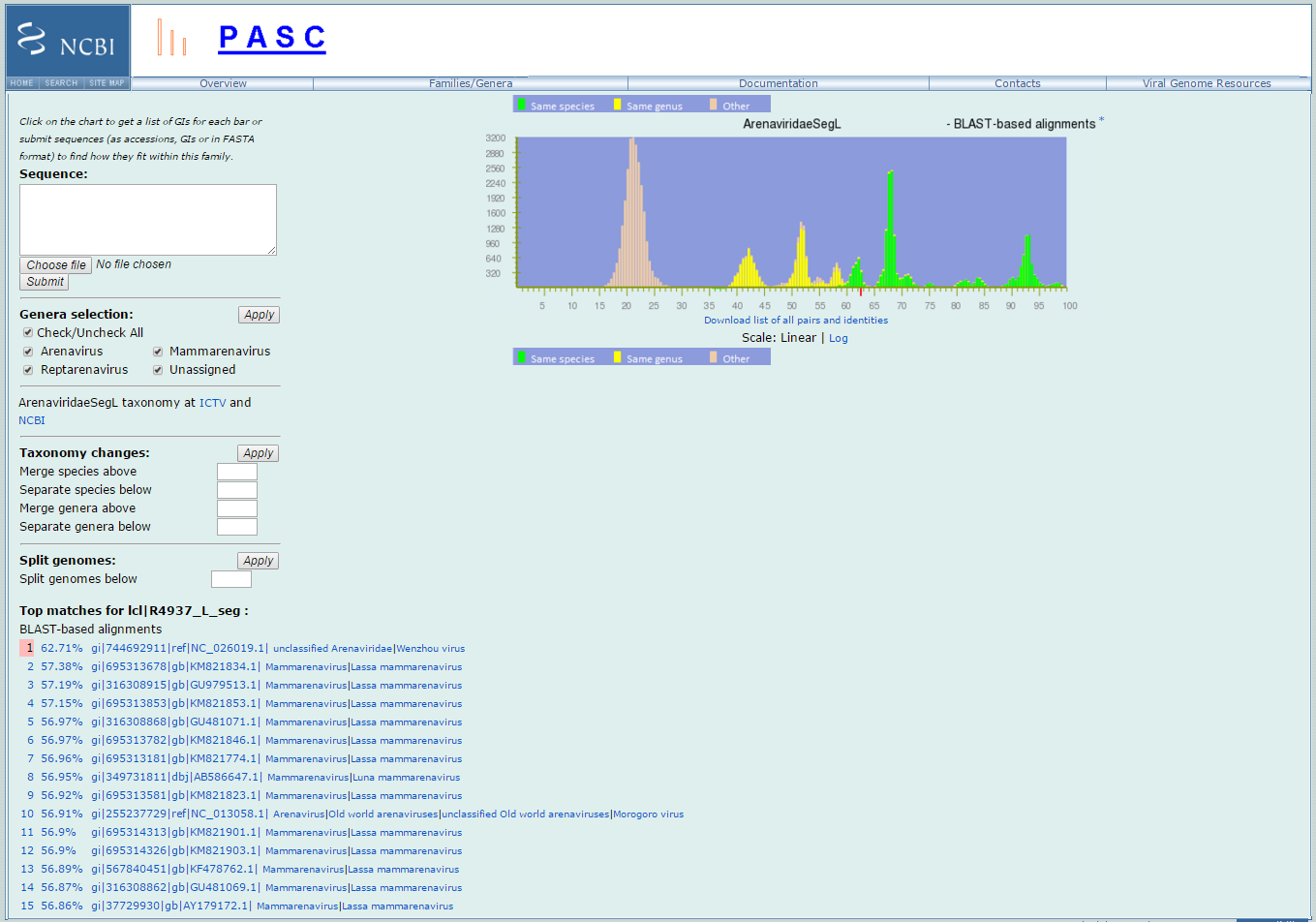


R4937: S segment


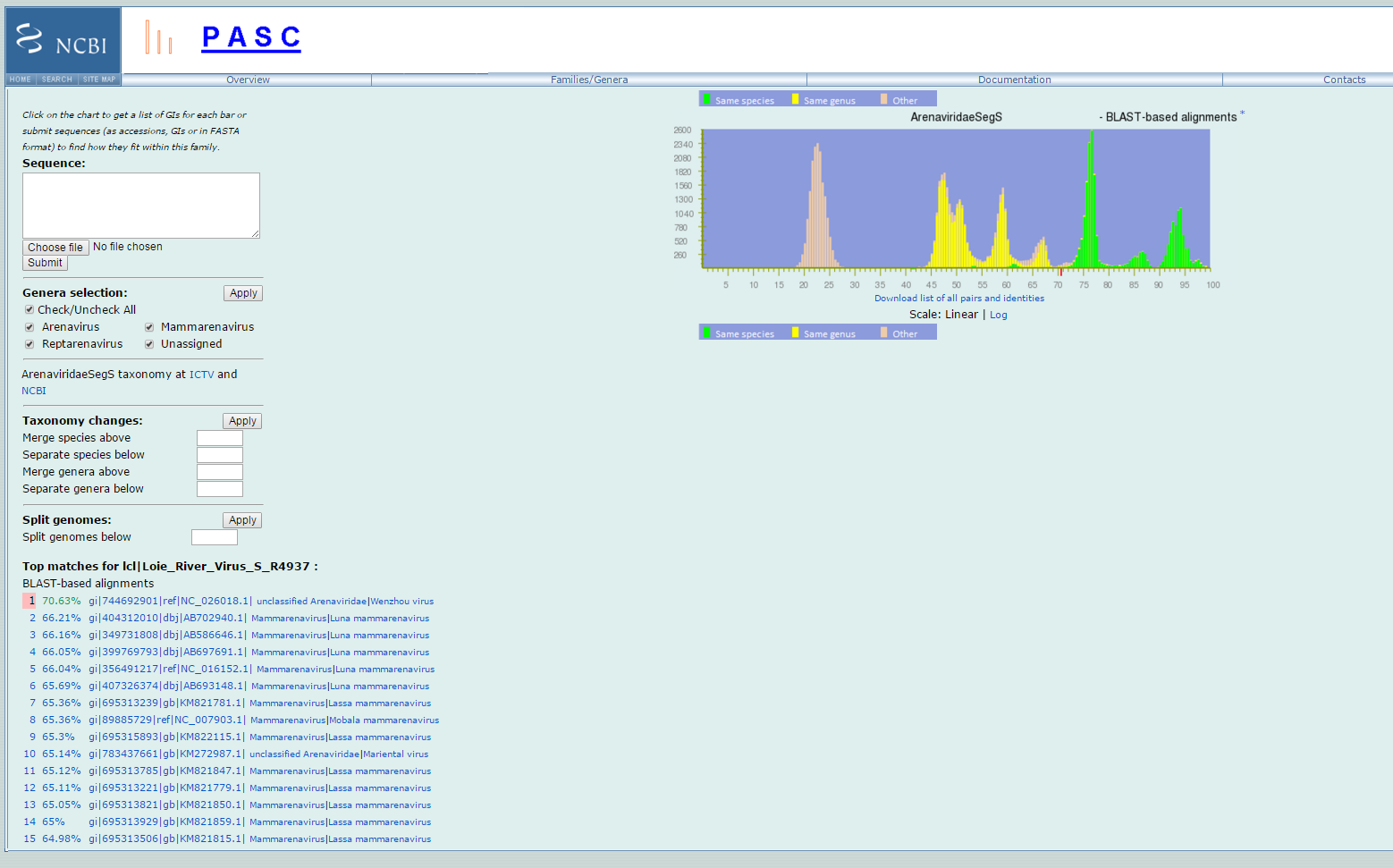


R5074: L segment


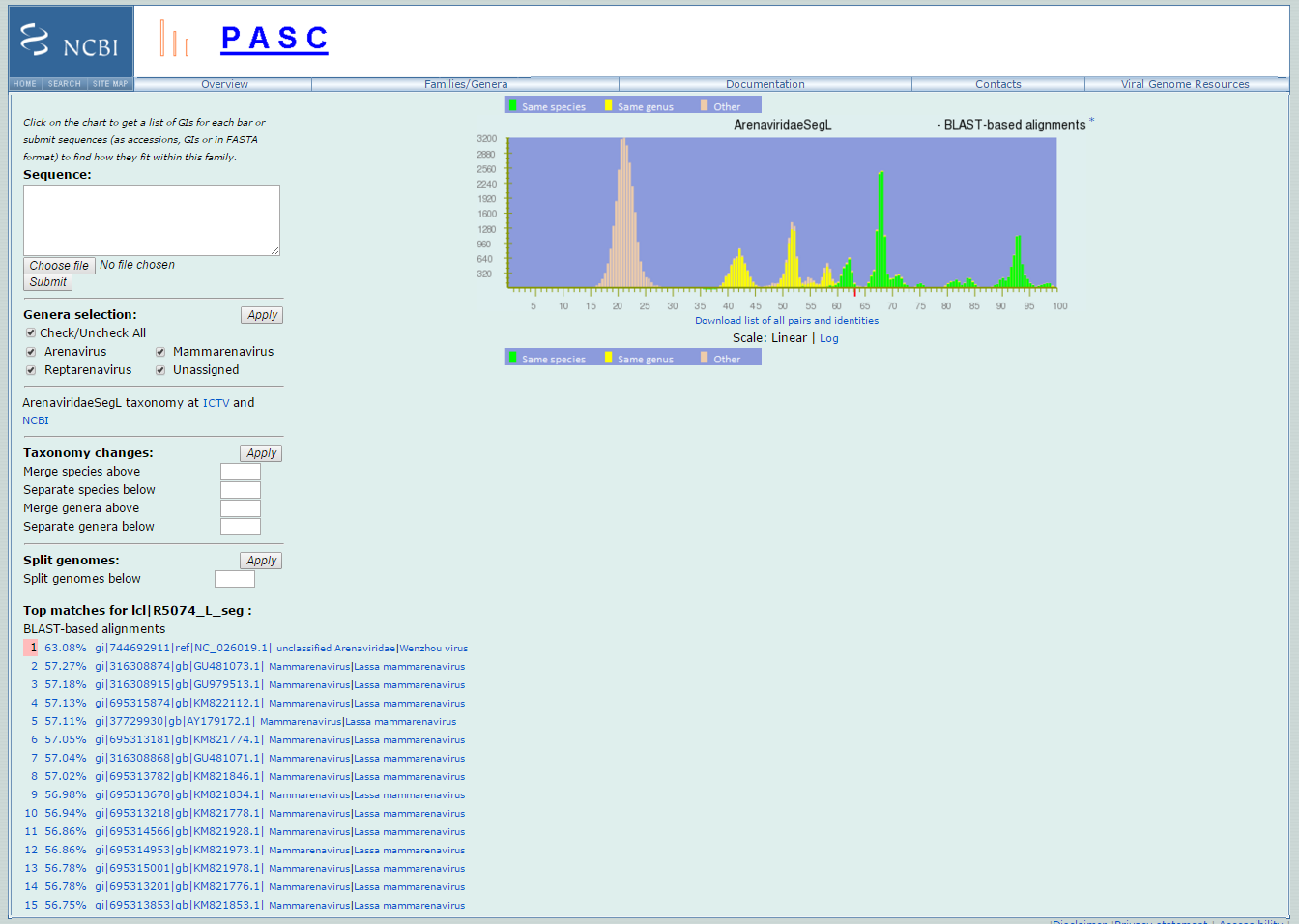


R5074: S segment


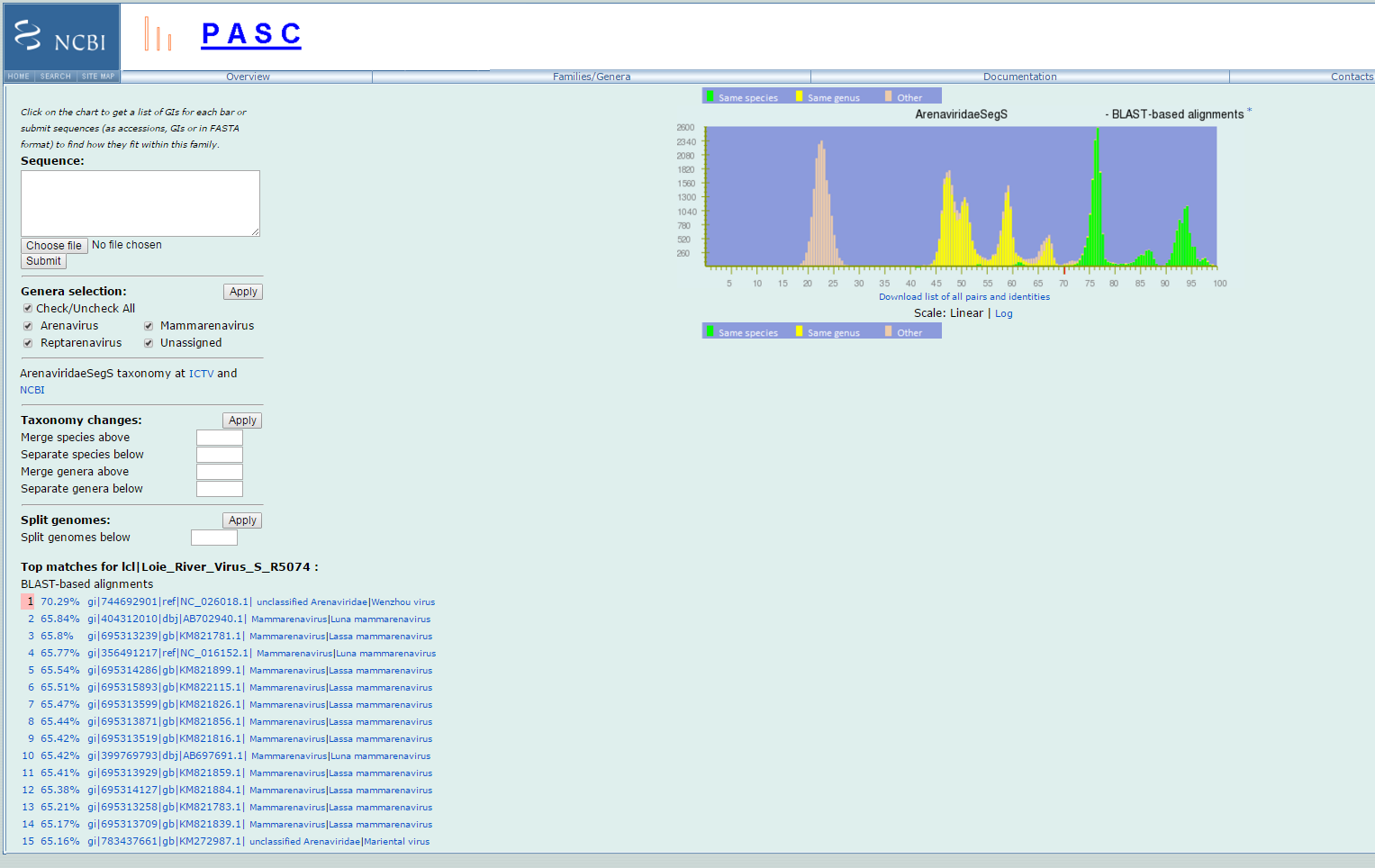

Supplement: Table 2—source data 1. — DOI: http://dx.doi.org/10.7554/eLife.13135.005 [file elife-13135-table2-data1.docx]
