# Supplementary material for: Evidence of human infection by a new mammarenavirus endemic to Southeastern Asia
Source: eLife. 2016 Jun 9;5:e13135. doi: 10.7554/eLife.13135 (PMC4900801; doi:10.7554/eLife.13135)
Supplement: Supplementary file 1. — (A) Details of primers used for diagnostic RT-PCRs and genome sequencing. (B) Animals tested for arenavirus RNA by species and site, with number of positives shown in bold. (C) Details of rodent samples positive for arenavirus infection by screening RT-PCR .(D) Details of animals used for Cambodian virus infections. (E) Detailed IgG ELISA results for 7 patients with seroconversion. (F) Comparison between IFA and IgG ELISA results in human sera. (G) Arenavirus infections in patients with ILI symptoms who tested negative for 4 common respiratory viruses versus patients with ILI symptoms who tested positive and control group of healthy individuals. (H) Statistic analysis by age between respiratory illness group and healthy control group. (I) Multiple alignment of the sequences of the amplicons obtained by PCR for 3 patients. (J) Results of IgG ELISA in experimentally-infected rodents. DOI: http://dx.doi.org/10.7554/eLife.13135.013 [file elife-13135-supp1.docx]

**Supplementary file 1A | Details of primers used for diagnostic RT-PCRs and genome sequencing**

| Primer name | Sequence | | | Target gene/ region | Method | | | Reference |
| --- | --- | --- | --- | --- | --- | --- | --- | --- |
| LVL3359plus | AGAATYAGTGAAAGGGARAGCAATTC | | | L | Screening RT-PCR  Screening RT-PCR | | | Vieth et al. 2007 |
| LVL3754minus | CACATCATTGGTCCCCATTTACTRTGATC | | | L |  |  |  | Vieth et al. 2007 |
| CAMN1 | TGGCTATCAGGGCATTCTCAAAC | | | L | Screening nested RT-PCR | | | Vieth et al. & this study |
| ARV2747-F | CCATCWACCCARTCYTTRACATC | | |  | Semi-nested RT-PCR & qRT-PCR | | | This study |
| ARV-2937-R | AARTGYAAAGARCCVCATCA | | |  | Semi nested RT-PC & qRT-PCR | | | This study |
| ARV3012-R | AAATCAAGRTTYTWTGTYTGGG | | |  | Semi-nested RT-PCR | | | This study |
| ARV-probe | 5’-FAM-ACACCATTNGCMACHGAYTGATC-3’-BHQ1 | | |  | qRT-PCR | | | This study |
| M13-M19C | TGTAAAACGGCCAGTGCGCACAGTGGATCCTAGGC | | | Terminal ends of both S and L segments | Whole S & L segment amplification | | | Bowen et al. 2000 |
| M13F | GTAAAACGACGGCCAG | | | TOPO plasmid | Whole S segment sequencing | | | TOPO^®^ TA cloning kit |
| M13R | CAGGAAACAGCTATGAC | | | TOPO plasmid |  |  |  | TOPO^®^ TA cloning kit |
| ARVAS_511F | CAGTATGARGCYATGAGY | | | GPC |  |  |  | This study |
| ARVAS_740F | TAATACAGAACACAACWTGGGR | | | GPC |  |  |  | This study |
| ARVAS_740R | YCCCAWGTTGTGTTCTGTATTA | | | GPC |  |  |  | This study |
| ARVAS_1606F | ACGGGGGCACTCTGTTTACA | | | N |  |  |  | This study |
| ARVAS_2341F | CAGTGTTGTCCCARGCYC | | | N |  |  |  | This study |
| ARVAS_2341R | GRGCYTGGGACAACACTG | | | N |  |  |  | This study |
| ARVAS_2799R | CAGTTYGGCACAATGCCCAGYCT | | | N |  |  |  | This study |
| R_C14_400 | AGCTTTGTTGGTAAGGGCAGCTTGC | | | L | Whole L segment sequencing | | | This study |
| F_B1_1551 | AGAGAACCCTGAGCTCATGTCGTA | | | L |  |  |  | This study |
| R_C2_2135 | AGGAACAAGCGCAGATGTGA | | | L |  |  |  | This study |
| F_C3_2056 | TGGTTCATGCTCACCTTATCTTC | | | L |  |  |  | This study |
| R_C3_2781 | GGAATTACACGAAGAGTTTACAGC | | | L |  |  |  | This study |
| F_C4_2413 | CTCACACACTTGCTCCCCAGTGT | | | L |  |  |  | This study |
| R_C4_3077 | AAGTGTAAAGAACCCCATCAACTGG | | | L |  |  |  | This study |
| F_C5_3005 | GGACACCATTTGCCACAGACTG | | | L |  |  |  | This study |
| R_C5_3744 | ATACAGCCTGGACCACAGCAAGTG | | | L |  |  |  | This study |
| F_C6_3549 | CCTCATCATTGCGGAGATCACATTG | | | L |  |  |  | This study |
| R_C6_4269 | GATTAGTAAGAGTGTGGCAAGTCGG | | | L |  |  |  | This study |
| F_C7_3970 | CCAACAGATGTGAAAGACTCTGG | | | L |  |  |  | This study |
| R_C7_4761 | GGCAAGTACCTATTGAACAAGGATGA | | | L |  |  |  | This study |
| F_C8_4564 | GTCCACCCTTTGCTTGACACAA | | | L |  |  |  | This study |
| R_C8_5118 | GATGTGATCACAGATGATGAGTTGG | | | L |  |  |  | This study |
| F_C9_4963 | GCCTGCGTTGAATGATGAAACC | | | L |  |  |  | This study |
| R_C9_5694 | CAGAAGACAGGTGAATCTTCCAAGTG | | | L |  |  |  | This study |
| F_C10_5526 | GCTCTTCACATCCATCCAACC | | | L |  |  |  | This study |
| R_C10_6097 | CTTCCAAGACATGATGAAGAACACC | | | L |  |  |  | This study |
| F_C11_5954 | CCATCTTTCCTGAAGGCCATTAGCT | | | L |  |  |  | This study |
| R_C11_6694 | CAGCTCGAGAGATAAGGGAGAAGG | | | L |  |  |  | This study |
| F_C12_6602 | CCTTAGCGAAGTGGTGCTTAATTGTC | | | L |  |  |  | This study |
| R_C12_7075 | GCTTTGTAAGAAGTAGCCCAGC | | | L |  |  |  | This study |
| F_C13_6955 | CCATCCACAATGGGGATGAGTTGAA | | | L |  |  |  | This study |
|  | |  |  | | |  |  |  |
|  | |  |  |  |  |  |  |  |

**Supplementary file 1B |** **Animals tested for arenavirus RNA by species and site, with number of positives shown in bold**

| Animal (species) | Thailand | | | Lao PDR | | Cambodia | | Total |
| --- | --- | --- | --- | --- | --- | --- | --- | --- |
|  | **Buriram** | **Loei** | **Nan** | **Luang Prabang** | **Pakse** | **Mondulkiri** | **Veal Renh** |  |
| Greater bandicoot rat (*Bandicota indica*) | 0 | 12/**3** | 17 | 1 | 1 | 0 | 0 | 31/**3** |
| Savile’s bandicoot rat (*Bandicota savilei*) | 2 | 21/**6** | 0 | 0 | 1 | 11 | 0 | 35/**4** |
| Berdmore’s berylmys (*Berylmys berdmorei*) | 0 | 3 | 0 | 0 | 0 | 0 | 6 | 9 |
| Bower’s berylmys (*Berylmys bowersi*) | 0 | 11 | 0 | 1 | 0 | 0 | 0 | 12 |
| Indomalayan pencil-tailed tree mouse (*Chiropodomys gliriodes*) | 0 | 1 | 0 | 0 | 0 | 0 | 0 | 1 |
| Asian gray shrew (*Crocidura attenuata*) | 0 | 1 | 0 | 0 | 0 | 0 | 0 | 1 |
| Edward’s leopoldamys (*Leopoldamys edwardsi*) | 0 | 2 | 0 | 0 | 0 | 0 | 0 | 2 |
| Indomalayan leopoldamys (*Leopoldamys sabanus*) | 0 | 2 | 0 | 0 | 0 | 0 | 0 | 2 |
| Indomalayan maxomys (*Maxomys surifer*) | 0 | 9 | 0 | 0 | 0 | 2 | 11 | 22 |
| Ryukyu mouse (*Mus caroli*) | 4 | 9 | 0 | 0 | 0 | 0 | 0 | 13 |
| Fawn-colored mouse (*Mus cervicolor*) | 2 | 17 | 1 | 2 | 0 | 0 | 0 | 22 |
| Cook’s mouse (*Mus cookii*) | 0 | 8 | 0 | 1 | 0 | 0 | 0 | 14 |
| Indomalayan niviventer (*Niviventer fulvescens*) | 0 | 53/**1** | 0 | 0 | 0 | 0 | 2 | 55/**1** |
| Ricefield rat (*Rattus argentiventer*) | 0 | 0 | 0 | 0 | 0 | 0 | 8 | 8 |
| Pacific rat (*Rattus exulans*) | 72 | 25 | 5 | 0 | 26 | 37 | 89/**13** | 254/**13** |
| Losea rat (*Rattus losea*) | 0 | 33 | 0 | 0 | 0 | 0 | 0 | 33 |
| White-footed Indochinese rat (*Rattus nitidius*) | 0 | 0 | 0 | 5 | 0 | 0 | 0 | 5 |
| Brown rat (*Rattus norvegicus*) | 0 | 0 | 0 | 0 | 0 | 0 | 19/**4** | 19/**4** |
| Oriental house rat (*Rattus tanezumi*) | 1 | 7 | 7 | 8 | 0 | 8 | 19 | 50 |
| Asian house shrew (*Suncus murinus*) | 0 | 0 | 0 | 0 | 0 | 0 | 39 | 39 |
| Total | 81 | 214 | 30 | 23 | 28 | 58 | 193 | 627/**27** |

**Supplementary file 1C |** **Details of rodent samples positive for arenavirus infection by screening RT-PCR**

| ID | Animal (species) | Date caught | Habitat type | Trapping site | Country |
| --- | --- | --- | --- | --- | --- |
| C0210 | Brown rat (*Rattus norvegicus*) | 26/11/2008 | Isolated settlement alongside canal | Veal Renh | Cambodia |
| C0232 | Pacific rat (*Rattus exulans*) | 27/11/2008 | Isolated settlement |  |  |
| C0253 | Pacific rat (*Rattus exulans*) | 28/11/2008 | Village |  |  |
| C0283 | Pacific rat (*Rattus exulans*) | 29/11/2008 | Village |  |  |
| C0605 | Brown rat (*Rattus norvegicus*) | 09/07/2009 | Hunter, unknown |  |  |
| C0617 | Brown rat (*Rattus norvegicus*) | 09/07/2009 | Village |  |  |
| C0621 | Pacific rat (*Rattus exulans*) | 09/07/2009 | Village |  |  |
| C0623 | Pacific rat (*Rattus exulans*) | 09/07/2009 | Village |  |  |
| C0639 | Pacific rat (*Rattus exulans*) | 10/07/2009 | Village |  |  |
| C0649 | Pacific rat (*Rattus exulans*) | 10/07/2009 | Village |  |  |
| C0650 | Pacific rat (*Rattus exulans*) | 10/07/2009 | Village |  |  |
| C0651 | Pacific rat (*Rattus exulans*) | 10/07/2009 | Village |  |  |
| C0661 | Pacific rat (*Rattus exulans*) | 11/07/2009 | Village |  |  |
| C0663 | Pacific rat (*Rattus exulans*) | 11/07/2009 | Village |  |  |
| C0667 | Brown rat (*Rattus norvegicus*) | 11/07/2009 | Village |  |  |
| C0671 | Pacific rat (*Rattus exulans*) | 11/07/2009 | Village |  |  |
| C0680 | Pacific rat (*Rattus exulans*) | 11/07/2009 | Village |  |  |
| R4831 | Great bandicoot rat (*Bandicota indica*) | 12/02/2008 | Lowland soybean plantation | Loei | Thailand |
| R4866 | Savile’s bandicoot rat (*Bandicota savilei*) | 13/02/2008 | Lowland soybean plantation |  |  |
| R4868 | Savile’s bandicoot rat (*Bandicota savilei*) | 13/02/2008 | Lowland soybean plantation |  |  |
| R4919 | Savile’s bandicoot rat (*Bandicota savilei*) | 14/02/2008 | Lowland soybean plantation |  |  |
| R4937 | Great bandicoot rat (*Bandicota indica*) | 15/02/2008 | Hunter, unknown |  |  |
| R4961 | Savile’s bandicoot rat (*Bandicota savilei*) | 16/02/2008 | Hunter, unknown |  |  |
| R4977 | Great bandicoot rat (*Bandicota indica*) | 16/02/2008 | Hunter, unknown |  |  |
| R4990 | Savile’s bandicoot rat (*Bandicota savilei*) | 16/02/2008 | Hunter, unknown |  |  |
| R5074 | Savile’s bandicoot rat (*Bandicota savilei*) | 18/02/2008 | Lowland soybean plantation |  |  |
| R5167 | Indomalayan niviventer (*Niviventer fulvescens*) | 20/02/2008 | Hunter, unknown |  |  |

**Supplementary file 1D | Details of animals used for Cambodian virus infections**

**a. Duration of infection experiment**

| Animal ID | Sex | Inoculated with infected tissues | Day killed post inoculation (or birth if applicable) | Organ(s) viral RNA positive | Seroconversion | Infection status |
| --- | --- | --- | --- | --- | --- | --- |
| **Wistar laboratory rats (adult)** | | | | | | |
| RNAD1 | M | Yes | 3 | Yes | Untested | Positive |
| RNAD2 | M | Yes | 5 | No | Untested | Unknown |
| RNAD3 | M | Yes | 7 | Yes | Untested | Positive |
| RNAD4 | M | Yes | 11 | No | Yes | Positive |
| RNAD5 | M | Yes | 11 | Yes | Yes | Positive |
| RNAD6 | M | Yes | 14 | Yes | Yes | Positive |
| RNAD7 | M | Yes | 14 | Yes | Yes | Positive |
| RNAD8 | F | Yes | 15 | Yes | Yes | Positive |
| RNAD9 | F | Yes | 15 | Yes | Yes | Positive |
| RNAD10 | M | Yes | 15 | No | No | Negative |
| RNAD11 | M | Yes | 15 | No | Yes | Positive |
| RNAD12 | M | Yes | 20 | No | Yes | Positive |
| RNAD13 | M | Yes | 20 | Yes | Yes | Positive |
| RNAD14 | F | Yes | 22 | Yes | Yes | Positive |
| RNAD15 | F | Yes | 22 | Yes | Yes | Positive |
| RNAD16 | M | Yes | 26 | No | Yes | Positive |
| RNAD17 | M | Yes | 28 | No | Yes | Positive |
| RNAD18 | M | Yes | 40 | Yes | Yes | Positive |
| RNAD19 | M | Yes | 40 | Yes | Yes | Positive |
| RNADN1 | F | No | 22 | No | No | Negative |
| RNADN2 | F | No | 40 | No | No | Negative |
| RNAD0A | M | Yes | 0 | Yes | No | Positive |
| RNAD0B | F | Yes | 0 | Yes | No | Positive |
| RNAD7A | M | Yes | 7 | Yes | No | Positive |
| RNAD7B | F | Yes | 7 | Yes | Yes | Positive |
| RNAD14A | M | Yes | 14 | Yes | Yes | Positive |
| RNAD14B | F | Yes | 14 | Yes | Yes | Positive |
| RNAD21A | M | Yes | 21 | Yes | Yes | Positive |
| RNAD21B | F | Yes | 21 | Yes | Yes | Positive |
| RNAD28A | M | Yes | 28 | Yes | Yes | Positive |
| RNAD28B | F | Yes | 28 | Yes | Yes | Positive |
| RNAD35A | M | Yes | 35 | Yes | Yes | Positive |
| RNAD35B | F | Yes | 35 | Yes | Yes | Positive |
| RNAD42A | M | Yes | 42 | Yes | Yes | Positive |
| RNAD42B | F | Yes | 42 | Yes | Yes | Positive |
| RNAD49A | M | Yes | 49 | Yes | Yes | Positive |
| RNAD49B | F | Yes | 49 | Yes | Yes | Positive |
| RNAD56A | M | Yes | 56 | Yes | Yes | Positive |
| RNAD56B | F | Yes | 56 | Yes | Yes | Positive |
| RNADN3 | M | No | 32 | No | No | Negative |
| RNADN4 | F | No | 32 | No | No | Negative |
| RNADN5 | M | No | 32 | No | No | Negative |
| RNADN6 | F | No | 32 | No | No | Negative |
| RNADN7 | M | No | 32 | No | No | Negative |
| RNADN8 | F | No | 32 | No | No | Negative |
| RNADN9 | M | No | 32 | No | No | Negative |
| RNADN10 | F | No | 32 | No | No | Negative |
| RNADN11 | M | No | 32 | No | No | Negative |
| RNADN12 | F | No | 32 | No | No | Negative |
| RNADN13 | M | No | 32 | No | No | Negative |
| RNADN14 | F | No | 32 | No | No | Negative |
| RNADN15 | M | No | 32 | No | No | Negative |
| RNADN16 | F | No | 32 | No | No | Negative |
| RNADN17 | M | No | 32 | No | No | Negative |
| **Pacific rats (adult)** | | | | | | |
| READ1 | M | Yes | 3 | No | Untested | Unknown |
| READ2 | M | Yes | 3 | Yes | Untested | Positive |
| READ3 | M | Yes | 7 | No | Untested | Negative |
| READ4 | M | Yes | 7 | Yes | Untested | Positive |
| READ5 | M | Yes | 11 | Yes | Yes | Positive |
| READ6 | M | Yes | 11 | Yes | Yes | Positive |
| READ7 | M | Yes | 14 | Yes | Yes | Positive |
| READ8 | M | Yes | 15 | Yes | Yes | Positive |
| READ9 | M | Yes | 21 | Yes | Yes | Positive |
| READ10 | M | Yes | 21 | Yes | Yes | Positive |
| READ11 | M | Yes | 28 | Yes | Yes | Positive |
| READ12 | M | Yes | 28 | No | Yes | Positive |
| **Wistar laboratory rats (adult)** | | | | | | |
| RNADN3 | F | No | 32 | No | Yes | Positive |
| **Wistar laboratory rats (juvenile)** | | | | | | |
| RNJD1 | N/T | Yes | 7 | No | Untested | Negative |
| RNJD2 | N/T | Yes | 7 | Yes | Untested | Positive |
| RNJD3 | N/T | Yes | 14 | Yes | Untested | Positive |
| RNJD4 | N/T | Yes | 14 | Yes | Untested | Positive |
| RNJD5 | N/T | Yes | 28 | Yes | Untested | Positive |
| RNJD6 | N/T | Yes | 28 | Yes | Untested | Positive |
| RNJD7 | N/T | Yes | 32 | Yes | Untested | Positive |
| RNJD8 | N/T | Yes | 32 | Yes | Untested | Positive |
| RNJD9 | N/T | Yes | 32 | Yes | Untested | Positive |
| RNJD10 | N/T | Yes | 32 | Yes | Untested | Positive |
| **Wistar laboratory rats (adult)** | | | | | | |
| RNADN4 | F | No | 32 | Yes | Yes | Positive |
| **Wistar laboratory rats (juvenile)** | | | | | | |
| RNJD11 | N/T | Yes | 7 | Yes | Untested | Positive |
| RNJD12 | N/T | Yes | 7 | Yes | Untested | Positive |
| RNJD13 | N/T | Yes | 14 | Yes | Untested | Positive |
| RNJD14 | N/T | Yes | 14 | Yes | Untested | Positive |
| RNJD15 | N/T | Yes | 28 | Yes | Untested | Positive |
| RNJD16 | N/T | Yes | 28 | Yes | Untested | Positive |
| RNJD17 | N/T | Yes | 32 | Yes | Untested | Positive |
| RNJD18 | N/T | Yes | 32 | Yes | Untested | Positive |
| RNJD19 | N/T | Yes | 32 | Yes | Untested | Positive |
| **Wistar laboratory rats (adult)** | | | | | | |
| READN1 | F | No | 32 | Yes | No | Positive |
| **Wistar laboratory rats (juvenile)** | | | | | | |
| REJD1 | N/T | Yes | 14 | No | Untested | Negative |
| REJD2 | N/T | Yes | 28 | Yes | Untested | Positive |
| REJD3 | N/T | Yes | 32 | No | Untested | Negative |

**b. Horizontal transmission experiment**

| Animal ID | Sex | Inoculated with infected tissues | Day killed post inoculation (or birth if applicable) | Paired individual/ dam | Organ(s) viral RNA positive | Seroconversion | Infection status |
| --- | --- | --- | --- | --- | --- | --- | --- |
| **Wistar laboratory rats (adult)** | | | | | | | |
| RNAH1 | M | Yes | 28 | RNAHU1 | No | Yes | Positive |
| RNAHU1 | M | No | 28 | RNAH1 | No | Negative | Negative |
| RNAH2 | M | Yes | 28 | RNAHU2 | No | Yes | Positive |
| RNAHU2 | M | No | 28 | RNAH2 | No | Negative | Negative |
| RNAH3 | M | Yes | 28 | RNAHU3 | No | Yes | Positive |
| RNAHU3 | M | No | 28 | RNAH3 | No | Negative | Negative |
| RNAH4 | M | Yes | 28 | RNAHU4 | No | Yes | Positive |
| RNAHU4 | M | No | 28 | RNAH4 | No | Negative | Negative |
| RNAH5 | M | Yes | 28 | RNAHU5 | No | Yes | Positive |
| RNAHU5 | M | No | 28 | RNAH5 | No | Negative | Negative |
| RNAH6 | M | Yes | 28 | RNAHU6 | No | Yes | Positive |
| RNAHU6 | M | No | 28 | RNAH6 | No | Negative | Negative |
| RNAH7 | M | Yes | 31 | RNAHU7 | No | Negative | Negative |
| RNAHU7 | M | No | 31 | RNAH7 | No | Negative | Negative |
| RNAH8 | M | Yes | 31 | RNAHU8 | No | Yes | Positive |
| RNAHU8 | M | No | 31 | RNAH8 | No | Negative | Negative |
| RNAHN1 | M | No | 31 | RNAHN2 | No | Negative | Negative |
| RNAHN2 | M | No | 31 | RNAHN1 | No | Negative | Negative |

**c. Vertical transmission experiment**

| Animal ID | Age | Sex | Inoculated with infected tissues | Day killed post inoculation (or birth if applicable) | Day born post inoculation | Paired individual/dam | Organ(s) viral RNA positive | Seroconversion | Infection status |
| --- | --- | --- | --- | --- | --- | --- | --- | --- | --- |
| **Wistar laboratory rats** | | | | | | | | | |
| RNV1 | Adult (Dam N1) | F | Yes | 26 | N/A | N/A | Yes | Yes | Positive |
| RNV1a | Juvenile | N/T | No | 26 (8) | 18 | RNV1 | No | Yes (maternal) | Negative |
| RBV1b |  | N/T | No | 26 (8) |  |  | No | Yes (maternal) | Negative |
| RNV2 | Adult (Dam N2) | F | Yes | 28 | N/A | N/A | No | Yes | Positive |
| RNV2a | Juvenile | N/T | No | 7 (0) | 7 | RNV2 | No | Untested | Negative |
| RNV2b |  | N/T | No | 9 (2) |  |  | No | Untested | Negative |
| RNV2c |  | N/T | No | 11 (4) |  |  | No | Untested | Negative |
| RNV2d |  | N/T | No | 13 (6) |  |  | No | Untested | Negative |
| RNV2e |  | N/T | No | 15 (8) |  |  | No | Untested | Negative |
| RNV2f |  | N/T | No | 17 (10) |  |  | No | Untested | Negative |
| RNV2g |  | N/T | No | 19 (12) |  |  | No | Untested | Negative |
| RNV2h |  | N/T | No | 21 (14) |  |  | No | Untested | Negative |
| RNV2i |  | N/T | No | 28 (21) |  |  | No | Yes (maternal) | Negative |
| RNV2j |  | N/T | No | 28 (21) |  |  | No | Yes (maternal) | Negative |
| RNV3 | Adult (Dam N3) | F | Yes | 28 | N/A | N/A | Yes | Yes | Positive |
| RNV3a | Juvenile | N/T | No | 7 (1) | 6 | RNV3 | No | Untested | Negative |
| RNV3b |  | N/T | No | 16 (10) |  |  | No | Untested | Negative |
| RNV3c |  | N/T | No | 28 (12) |  |  | No | Untested | Negative |
| RNV4 | Adult (Dam N4) | F | Yes | 10 | N/A | N/A | Yes | Yes | Positive |
| RNV4a | Juvenile | N/T | No | 3 (1) | 2 | RNV4 | No | Untested | Negative |
| RNV4b |  | N/T | No | 3 (1) |  |  | No | Untested | Negative |
| **Pacific rats** | | | | | | | | | |
| REV1 | Adult (Dam E1) | F | Yes | 31 | N/A | N/A | No | Yes | Positive |
| REV1a | Juvenile | N/T | No | 1 (0) | 1 | REV1 | No | Untested | Negative |
| REV1b |  | N/T | No | 3 (2) |  |  | No | Untested | Negative |
| REV1c |  | N/T | No | 7 (6) |  |  | Yes | Untested | Positive |
| REV1d |  | N/T | No | 21 (20) |  |  | No | Untested | Negative |
| REV1e |  | N/T | No | 50 (49) |  |  | No | Untested | Negative |
| REV2 | Adult (Dam E2) | F | Yes | 37 | N/A | N/A | Yes | Yes | Positive |
| REV2a | Juvenile | N/T | No | 2 (1) | 1 | REV2 | No | Untested | Negative |
| REV2b |  | N/T | No | 10 (9) |  |  | No | Untested | Negative |
| REV2c |  | N/T | No | 37 (36) |  |  | Yes | Untested | Positive |

Legend: As the age of wild-caught animals was unknown, weaned individuals or those >60 days old were considered as adults, whilst younger individuals were considered as juveniles; the sex of juveniles was not confirmed = N/T. Maternal refers to the presence of antibodies potentially of maternal origin.

**Supplementary file 1E | Detailed IgG ELISA results for 7 patients with seroconversion.**

| **Patient ID** | **Date of sampling S1** | **S1 IgG OD** | **S1 IgG result** | **Interval S1-S2 (days)** | **Date of sampling S2** | **S2 IgG OD** | **S2 IgG result** | **S1 50%OD** | **OD S2 - OD S1** |
| --- | --- | --- | --- | --- | --- | --- | --- | --- | --- |
| KP437 | 9-juil-09 | 0,14 | Negative | 9 | 18-Jul-09 | 0,65 | Positive | 0,07 | 0,51 |
| KH4 | 19-Apr-10 | 0,19 | Negative | 15 | 4-May-10 | 0,28 | Positive | 0,09 | 0,09 |
| Q1019201 | 17-Oct-06 | 0,24 | Positive | 7 | 24-Oct-06 | 0,48 | Positive | 0,12 | 0,24 |
| R0627064 | 17-Jun-07 | 0,36 | Positive | 8 | 25-Jun-07 | 1,24 | Positive | 0,18 | 0,89 |
| S0626203 | 25-06-2008 | 0,31 | Positive | 5 | 30-Jun-08 | 0,65 | Positive | 0,15 | 0,34 |
| S0723092 | 23-Jul-08 | 0,17 | Negative | 6 | 29-Jul-08 | 0,37 | Positive | 0,09 | 0,19 |
| S0911085 | 10-Sep-08 | 0,33 | Positive | 2 | 12-Sep-08 | 0,85 | Positive | 0,17 | 0,52 |

Legend: OD: optical density; S1: first serum sample collected (during the acute phase of the disease); S2: Second serum sample collected (during the convalescent phase of the disease)

**Supplementary file 1F | Comparison between IFA and IgG ELISA results in human sera.**

|  |  | **ELISA** | |
| --- | --- | --- | --- |
|  |  | Negative | Positive |
| **IFA** | Negative | 351 | 11 |
|  | Positive | 2 | 8 |

| Spearman's Correlation = 0.565 | | |
| --- | --- | --- |
| p<0.001 |  |  |

**Supplementary file 1G | Arenavirus infections in patients with ILI symptoms who tested negative for 4 common respiratory viruses versus patients with ILI symptoms who tested positive and control group of healthy individuals**

|  | Patients with ILI symptoms who tested negative for influenza A virus, influenza B virus, human respiratory syncytial virus and human metapneumovirus (A)  N= 328 | Patients with ILI symptoms who tested positive for influenza A virus, influenza B virus, human respiratory syncytial virus and human metapneumovirus (B)  N=392 | Control group (C)  N=504 | P value |
| --- | --- | --- | --- | --- |
| Mean age (years)  (min-max) | 16.7  (0.1 – 82) | 10.3  (0.1 – 83) | 28  (0.8 – 83) | <0.0001  (A and B *vs* C) |
| Sex ratio | 0.53 | 0.50 | 0.52 | 0.648 |
| Arenavirus infections | 4 (1.2%) | 0 | 0 | 0.024 (A *vs* C)  0.043 (A *vs* B) |

Legend: ILI = Influenza-like Illness

**Supplementary file 1H | Statistic analysis by age between respiratory illness group and healthy control group**

| Age group (years) | Number | Dengue-like illness | Respiratory illness  [*number of arenavirus-positive cases*] | Healthy control | P value* |
| --- | --- | --- | --- | --- | --- |
| [0-5] | 661 | 82 | 530 [*3*] | 49 | p=0.597 |
| ]5-10] | 353 | 93 | 208 [*1*] | 52 | p=0.616 |
| ]10-15] | 205 | 62 | 108 | 35 | NA |
| ]15-20] | 67 | 4 | 25 | 38 | NA |
| ]20-30] | 132 | 2 | 19 | 111 | NA |
| ]30 | 317 | 2 | 106 [*2*] | 209 | p=0.046 |
| Total | 1735 | 245 | 996 [*6*]^#^ | 494^$^ | p<0.001 |

* Comparison between respiratory illness group and healthy control group

^#^ 3 without information on age

^$^ 10 without information on age

**Supplementary file 1I | Multiple alignment of the sequences of the amplicons obtained by PCR for 3 patients**

245 255 265 275 285 295

C649_Roden GGTGACTTCA AATCTATGTT CTGCACTAAC ATTAGAAATA AGAAAGGACA CATCATGGGT

P456_parti ---------- ---------- ---------- ---------- ---------A CATCATTGGT

P464_parti ---------- ---------- ---------- ---------- ---------- ----------

KP050_part ---------- ---------- ---------- ---------- ---------- ----------

....|....| ....|....| ....|....| ....|....| ....|....| ....|....|

305 315 325 335 345 355

C649_Roden CCCCACTTGC TGTGGTCCAG GCTGTATGAT AACTGAGCAA GAGACACAGA GAGTCTCATG

P456_parti CCCCATTTAC TATGATCCAG GCTGTATGAT AACTGAGCAA GAGACACAGA GAGTTTTTTG

P464_parti ---------- ---------- ---------- ---------- ---------- ----------

KP050_part ---------- ---------- ---------- ---------- ---------- ----------

....|....| ....|....| ....|....| ....|....| ....|....| ....|....|

365 375 385 395 405 415

C649_Roden GCTATCAGGG CATTCTCAAA CTCCTTTTCA TCATTCAAAC AACTCCCCCT GAAGTGTTTA

P456_parti GCTATCCAGG CATTCTCAAA CTCCTTTTCA TCATTCAAAC AACTCCCCCT GAAGTGTTTA

P464_parti ---------- ---------- ---------- ---------- ---------- ----------

KP050_part ---ATCAGGG CATTCTCAAA CTCCTTTTCA TCATTCAAAC AACTCCCCCT GAAGTGTTTA

....|....| ....|....| ....|....| ....|....| ....|....| ....|....|

425 435 445 455 465 475

C649_Roden GTGAATGCTT CAAAGTAATC TTCTATTAAC CTTGTGTACA TTTTGGTTCT GAGATCACCA

P456_parti GTGAATGCTT CAAAGTAATC TTCTATTAAC CTTGTGTACA TTTTGGTTCT GAGATCACCA

P464_parti ---------- ---------- ---------- ---------- ----GGTTCT GAGATCACCA

KP050_part GTGAATGCTT CAAAGTAATC TTCTATTAAC CTTGTGTACA TTTTGGTTCT GAGATCACCA

....|....| ....|....| ....|....| ....|....| ....|....| ....|....|

485 495 505 515 525 535

C649_Roden ATGTATAACT CCCGATTCCC TCCAACCTGC TCTTTGTAAG ACAACGAAAA CTTCAACCTT

P456_parti ATGTATAACT CCCGATTCCC TCCAACCTGC TCTTTGTAAG ACAACGAAAA CTTCAACCTT

P464_parti ATGTATAACT CCCGATTCCC TCCAACCTGC TCTTTGTAAG ACAACGAAAA CTTCAACCTT

KP050_part ATGTATAACT CCCGATTCCC TCCAACCTGC TCTTTGTAAG ACAACGAAAA CTTCAACCTT

....|....| ....|....| ....|....| ....|....| ....|....| ....|....|

545 555 565 575 585 595

C649_Roden CCGGTGTTTG GGCCAACAGA TGTGAAAGAC TCTGGTGACT CTTCACTATA AAAGCACAAG

P456_parti CCGGTGTTTG GGCCAACAGA TGTGAAAGAC TCTGGTGACT CTTCACTATA AAAGCACAAG

P464_parti CCGGTGTTTG GGCCAACAGA TGTGAAAGAC TCTGGTGACT CTTCACTATA AAAGCACAAG

KP050_part CCGGTGTTTG GGCCAACAGA TGTGAAAGAC TCTGGTGACT CTTCACTATA AAAGCACAAG

....|....| ....|....| ....|....| ....|....| ....|....| ....|....|

605 615 625 635 645 655

C649_Roden TTTTTTAAGG CCGCACTAGT GCAGTTTGAG AGGCTCAATG CCTTGCTTAG TGCCTCTGAA

P456_parti TTTTTTAAGG CCGCACTAGT GCAGTTTGAG AGGCTCAATG CCTTGCTTAG TGCCTCTGAA

P464_parti TTTTTTAAGG CCGCACTAGT GCAGTTTGAG AGGCTCAATG CCTTGCTTAG TGCCTCTGAG

KP050_part TTTTTTAAGG CCGCACTAGT GCAGTTTGAG AGGCTCAATG CCTTGCTTAG TGCCTCTGAG

....|....| ....|....| ....|....| ....|....| ....|....| ....|....|

665 675 685 695 705 715

C649_Roden TTGCTCTCCC TCTCACTGAT CCTGACATCA TCTAATAGCT TGCTATGATC AAATTTAAAA

P456_parti TTGCTTTCCC TT-------- ---------- ---------- ---------- ----------

P464_parti TTGCTTTCCC ---------- ---------- ---------- ---------- ----------

KP050_part TTGCTTTCCC TTTC------ ---------- ---------- ---------- ----------

....

C649 _Roden: sequence of the amplicon of the positive control used in all the series of PCRs and corresponding to the sample C649 of rodent origin detected in Cambodia. P4568_parti, P_464 parti, and KP050_parti correspond to the sequences of the amplicons generated by PCR for 3 Cambodian patients. Nucleotide differences with the sequence of the amplicon of rodent origin are highlighted in yellow for easier reading.

**Supplementary file 1J | Results of IgG ELISA in experimentally-infected rodents**

| **ID** | **Species** | **Date of inoculation** | **date of collection** | **IFA result** | **PCR result** | **OD IgG ELISA (cut off: 0.11)** | **ELISA result** |
| --- | --- | --- | --- | --- | --- | --- | --- |
| C649_A | *Rattus* norvegicus | 10-Aug-12 | Day 0 | NA |  | 0,07 | Negative |
| C649_A | *Rattus* norvegicus |  | Day 7 | NA |  | 0,19 | Positive |
| C649_A | *Rattus* norvegicus |  | Day 14 | NA |  | 0,46 | Positive |
| C649_A | *Rattus* norvegicus |  | Day 21 | NA |  | 0,504 | Positive |
| C649_A | *Rattus* norvegicus |  | Day 28 | NA |  | 0,52 | Positive |
| C649_A | *Rattus* norvegicus |  | Day 35 | NA |  | 0,57 | Positive |
| C649_A | *Rattus* norvegicus |  | Day 42 | NA |  | 0,63 | Positive |
| C649_A | *Rattus* norvegicus |  | Day 49 | NA |  | 0,64 | Positive |
| C649_A | *Rattus* norvegicus |  | Day 56 | NA | Positive in most organs | 0,55 | Positive |
| C649_B | *Rattus* norvegicus | 10-Aug-12 | Day 0 | NA |  | 0,07 | Negative |
| C649_B | *Rattus* norvegicus |  | Day 7 | NA |  | 0,044 | Negative |
| C649_B | *Rattus* norvegicus |  | Day 14 | NA |  | 0,45 | Positive |
| C649_B | *Rattus* norvegicus |  | Day 21 | NA |  | 0,57 | Positive |
| C649_B | *Rattus* norvegicus |  | Day 28 | NA |  | 0,55 | Positive |
| C649_B | *Rattus* norvegicus |  | Day 35 | NA |  | 0,57 | Positive |
| C649_B | *Rattus* norvegicus |  | Day 42 | NA |  | 0,6 | Positive |
| C649_B | *Rattus* norvegicus |  | Day 49 | NA |  | 0,56 | Positive |
| C649_B | *Rattus* norvegicus |  | Day 56 | NA | Positive in most organs | 0,7 | Positive |
| C649 2 | *Rattus norvegicus* | 17-May-10 | Day 15 | Positive | Positive in liver and spleen | 0,1275 | Positive |
| M649 A | *Rattus norvegicus* | 10-Jun-10 | Day 15 | Positive | Positive in lung | 0,1505 | Positive |
| A C649 | *Rattus norvegicus* | 4-Feb-10 | Day 0 | Negative |  | 0,002 | Negative |
| A C649 | *Rattus norvegicus* |  | Day 5 | Negative |  | 0,003 | Negative |
| A C649 | *Rattus norvegicus* |  | Day 8 | Negative |  | 0,084 | Negative |
| A C649 | *Rattus norvegicus* |  | Day 12 | Positive weak |  | 0,173 | Positive |
| A C649 | *Rattus norvegicus* |  | Day 15 | Positive | Positive in lung, heart and spleen | 0,375 | Positive |

Legend: OD: optical density
